# Supplementary material for: Reliable Prediction of Post-Operative Complications’ Rate Using the G8 Screening Tool: A Prospective Study on Elderly Patients Undergoing Surgery for Kidney Cancer
Source: J Clin Med. 2022 Jun 30;11(13):3785. doi: 10.3390/jcm11133785 (PMC9267910; doi:10.3390/jcm11133785)
Supplement: Supplementary file 1 [file jcm-11-03785-s001.zip › jcm-1746332-supplementary.pdf]

Table S1. Geriatric 8 (G8) Assessment Questionare

	Items	Possible answers (score)
**A**	Has food intake declined over the past 3 months due to loss of appetite, digestive problems, chewing or swallowing difficulties?	0 : severe decrease in food intake
		1 : moderate decrease in food intake
		2 : no decrease in food intake
**B**	Weight loss during the last 3 months	0 : weight loss > 3 kg
		1 : does not know
		2 : weight loss between 1 and 3 kg
		3 : no weight loss
**C**	Mobility	0 : bed or chair bound
		1 : able to get out of bed/chair but does not go out
		2 : goes out
**E**	Neuropsychological problems	0 : severe dementia or depression
		1 : mild dementia or depression
		2 : no psychological problems
**F**	Body Mass Index (BMI (weight in kg) / (height in m^2)	0 : BMI < 19
		1 : BMI = 19 to BMI < 21
		2 : BMI = 21 to BMI < 23
		3 : BMI = 23 and > 23
**H**	Takes more than 3 medications per day	0 : yes
		1 : no
**P**	In comparison with other people of the same age, how does the patient consider his/her health status?	0 : not as good
		0.5 : does not know
		1 : as good
		2 : better
	Age (years)	0 : >85
		1 : 80-85
		2 : <80
	**TOTAL SCORE**	**0 – 17**
